# Indications and outcome of pediatric tracheostomy: results from a Nigerian tertiary hospital

**DOI:** 10.1186/1471-2482-10-2

**Published:** 2010-01-12

**Authors:** Adeyi A Adoga, Nuhu D Ma'an

**Affiliations:** 1 Otorhinolaryngology unit, Department of Surgery, Jos University Teaching Hospital, PMB 2076, Jos, Plateau State, Nigeria

## Abstract

**Background:**

There is a change in the concept of pediatric tracheostomy. This study investigates the indications and outcomes of pediatric tracheostomy in a Nigerian teaching hospital finding out whether there is also a change in the trend in our environment as compared to other centers.

**Methods:**

A retrospective chart review of 46 patients aged between 2 months and 15 years who presented to our Otorhinolaryngological facility and had tracheostomy between January 2000 and December 2008.

**Results:**

The age range was 2 months to 15 years. There were 29 males and 17 females. Thirty two (69.6%) patients were in the age range 6-10 years. Forty tracheostomies (87%) were performed as emergency while 6 (13%) as elective procedures. The commonest indication for tracheostomy was upper airway obstruction (n = 29, 63%). Transverse skin incision was employed in all the cases. No intra-operative complication was recorded. The post-operative complication rate was 15.2%. The duration of tracheostomy ranged from 5 days to 3 months. All the patients were successfully decannulated. The overall mortality was 8 (17.4%). There was no tracheostomy related mortality.

**Conclusions:**

There is no increase in the incidence of tracheostomy in patients under 1 year of age and the commonest indication for the procedure in Nigeria has remained relief of upper airway obstruction. Pediatric tracheostomy is safe when performed in the tertiary hospital setting.

## Background

Tracheostomy in the pediatric age group is different from that in adults. In children it is a more laborious procedure with difficulties in post-operative management and it is commoner for children to suffer greater morbidity and mortality [1,2].

There is however a changing trend in the indications and outcomes in the use of tracheostomy in children for airway management [3-5]. In the past, the commonest indication was acute inflammatory airway obstruction [6] but in recent times, prolonged intubation has become the commonest indication [6,7]. Even the age at which tracheostomy is performed is becoming increasingly younger [8]. These have been attributed to the changes in the epidemiology of infectious diseases and the improvement in the capabilities of medical technology [9].

Is there a corresponding change in the indications and outcomes of pediatric tracheostomy in our environment? The literature is scarce on the experiences with tracheostomy in the pediatric age group in our environment.

The aim of this retrospective study is to highlight our experiences with pediatric patients who had tracheostomy between January 2000 and December 2008, comparing our results with those from other centers in the world.

## Methods

A retrospective chart review of 46 patients aged between 2 months and 15 years who presented to our Otorhinolaryngological facility and had tracheostomy between January 2000 and December 2008.

After obtaining clearance from the ethical committee of the Jos University Teaching Hospital, Jos, Nigeria, the medical records of these patients were retrieved and analyzed. The main parameters we studied were the age, gender, indications, surgical technique, complications and mortality rate.

## Results

Forty six pediatric patients had tracheostomy within the study period. The age range was 2 months to 15 years. There were 29 males and 17 females, giving a male to female ratio of 1.7:1. Thirty two (69.6%) patients were in the age range 6-10 years (Table 1).

**Table 1:** Age distribution of patients who had tracheostomy

Age (years)	Frequency	Percentage
0-5	10	21.7
6-10	32	69.6
11-15	4	8.7
**Total**	**46**	**100**

Forty tracheostomies (87%) were performed as an emergency while 6 (13%) as elective procedures.

The indications for tracheostomy (Table 2) were upper airway obstruction (n = 29, 63%), craniofacial trauma (n = 7, 15.2%), prolonged intubation (n = 5, 11%), infections (n = 2, 4.3%), head and neck malignancies (n = 2, 4.3%) and tracheobronchial toileting (n = 1, 2.2%). The commonest cause of upper airway obstruction requiring tracheostomy was respiratory papilloma (n = 20). At follow up, recurrence of respiratory papilloma occurred in 2 patients at 8 and 10 months respectively, each of whom had one repeated episode of direct laryngoscopy and clearance.

**Table 2:** Indications for tracheostomy

Indications	Frequency	Percentage
Upper airway obstruction	29	63
Craniofacial trauma	7	15.2
Prolonged intubation	5	11
Infections	2	4.3
Head and neck malignancies	2	4.3
Tracheobronchial toileting	1	2.2
**Total**	**46**	**100**

Horizontal skin incision was employed in all the cases.

**Figure 1:**
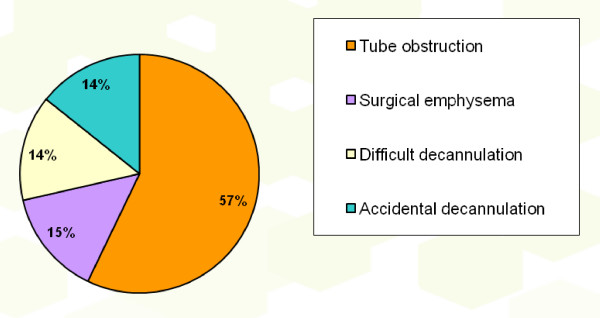
**Post-tracheostomy complications**.

No intra-operative complication was recorded. The post-operative complications seen (Figure 1) were tube obstruction (n = 4), surgical emphysema (n = 1), difficult decannulation (n = 1) and accidental decannulation (n = 1) giving a complication rate of 15.2%. No post-decannulation problems were encountered.

The duration of tracheostomy ranged from 5 days to 3 months. All the patients were successfully decannulated.

The overall mortality was 8 (17.4%). There was no tracheostomy related mortality. Follow up of all patients has been uneventful.

## Discussion

Tracheostomy is a surgical procedure in which an opening is made into the trachea and maintained with a tube in order to establish direct communication with the external environment.

It is not exempt from complications when performed in the pediatric age group. In fact, it is known to be associated with more morbidity and mortality in this age group [10]. The mortality figures recorded range from 0-6% [11]. However, adhering to the basic surgical technique and the avoidance of emergency tracheostomies as much as possible reduces the incidence of morbidities and mortalities that may follow.

In our series, majority (69.6%) of the patients who had tracheostomy was in the 6-10 age group and most of these patients were managed for recurrent respiratory papilloma. This is at variance with other reports which state that more tracheostomies were done for patients within the first year of life [12,13].

Males are more affected and this is because of their increased susceptibility to congenital and acquired disorders.

Many changes have occurred over the years in the use of pediatric tracheostomy for airway management. The indications and outcomes have changed with prolonged intubation being the commonest indication these days and the age at which tracheostomy is carried out is even becoming increasingly younger [6-8]. In the past, infective conditions such as epiglottitis and laryngotracheobronchitis were major indications for tracheostomy but the better handling of infections with the use of intubation and conservative management in the pediatric intensive care unit has reduced the incidence of these indications [1,9].

The commonest indication recorded in our series is upper airway obstruction primarily from recurrent respiratory papilloma, which necessitated emergency tracheostomy as these patients presented in respiratory distress as shown in a previous study from our center [14]. The high incidence of respiratory papilloma could be because of mother to child transmission of the Human Papilloma virus during delivery. Further research in our region is required to substantiate this. In a study of 58 children with recurrent respiratory papillomatosis in the United States, 12 (21%) had tracheostomies [15]. In our study, 2 patients with laryngotracheobronchitis presented in acute respiratory distress and had emergency tracheostomy to maintain airway and save their lives. It is not uncommon for patients in our environment to present very late to the hospital.

Craniofacial trauma formed 15.2% of the indications for the tracheostomy in this age group and interestingly all these injuries were from road traffic accidents especially involving motorcycles which have become a major means of commuter transportation in Nigeria. This is the leading cause of craniofacial injuries in our environment and children are not exempt from it. Recommendations have been given to those in government on ways of reducing road traffic accidents and these include the full enforcement of existing laws such as ensuring road worthy vehicles ply our roads, proper road maintenance and the enforcement of traffic rules and regulations especially the use of seat belts and helmets [16]. Our hope is that these recommendations will be implemented.

Prolonged intubation still forms a minor indication for pediatric tracheostomy in our environment. Most patients that may require prolonged intubation usually die from their ailments before tracheostomy is done.

The surgical technique employed in all our patients was the horizontal skin incision in the operating room. This is the method preferred by us whether it's an emergency or an elective tracheostomy because of the advantage of a better cosmetic result though, the vertical incision has the advantage of running in the line of the trachea and it is less vascular.

The procedure is done under general anesthesia via endotracheal intubation, laryngeal mask airway or facemask depending on the presentation of the patient who is placed supine with partial extension of the neck. Routine cleaning and draping is done and adrenaline (1:200,000 dilution) is injected into the skin of the anterior neck i.e. site of incision, midway between the cricoid cartilage and suprasternal notch. A horizontal 1.5 cm skin crease incision is made to the sub-platysmal level following which blunt dissection is continued vertically with an artery forceps in the midline as the assistant surgeon retracts the strap muscles until the trachea is approached. Bleeding is controlled by diathermy if necessary. The thyroid isthmus is freed and retracted superiorly or inferiorly, the fascia over the trachea is incised exposing the 2nd to 4th tracheal rings, a vertical incision is made through these rings. Blood and other secretions in the airway are suctioned if present and an appropriate sized tracheostomy tube is inserted and secured to the patient's neck. Postoperative care by suctioning secretions from the tube and close monitoring for complications such as surgical emphysema and pneumothorax is ensured.

We are aware of other techniques such as percutaneous dilatational tracheostomy (contraindicated in children) which has the advantage of reduced operation time and lower cost [17] but we lack the facilities in our center to perform such a procedure even in adult patients.

Pediatric tracheostomy is not bereft of complications. Intra and post-operative complications can be encountered with rates as high as 40% being reported [11]. A complication rate of 15.2% was recorded in our series. This is still lower than such reported cases. Tube obstruction occurred in 4 of our patients from dried crusts of mucous secretions. These were managed by instilling some drops of sodium bicarbonate solution and suctioning the tubes. The surgical emphysema noticed cleared on the 3^rd ^post-operative day. Difficult decannulation was encountered in 1 patient as a result of supra-stomal granulation tissue she developed necessitating tracheoscopy and excision before decannulation was effected successfully.

The decannulation method utilized was by occlusion of a smaller tracheostomy tube inserted after initial assessment of the child for the absence of aspiration during feeding and the absence of suprastomal granulation tissue by plain soft tissue neck X-ray. The child is observed during the day with the occluded tube and if well tolerated, the process is repeated during the night and removed if no problems.

The overall mortality recorded was 8 (17.4%) and these were from underlying diseases. Six of these patients died from thoraco-abdominal injuries sustained following road traffic accidents and 2 from the head and neck malignant conditions they presented with. These patients also presented late with advanced malignant tumors.

No tracheostomy related death occurred. This conforms to findings in other parts of the world, therefore indicating the safety of the procedure done in the tertiary hospital setting.

The limitation of this study is that it is retrospective from a single center with report from the experiences of three otolaryngologists. A prospective population based study may be helpful in order to determine the national incidence of pediatric tracheostomies.

## Conclusions

Even though we are still evolving in our otorhinolaryngological practice, we have not observed a decrease in the age group of patients requiring tracheostomy like in other centers in the world and the commonest indication for the procedure in Nigeria has remained relief of upper airway obstruction.

Pediatric tracheostomy is safe when performed in the tertiary hospital setting.

## Competing interests

The authors declare that they have no competing interests.

## Authors' contributions

AAA was the principal surgeon, performed literature search, prepared the manuscript, read and approved the final manuscript.

NDM assisted in the surgeries, post-operative management of patients, read and approved the final manuscript.

## Pre-publication history

The pre-publication history for this paper can be accessed here:

http://www.biomedcentral.com/1471-2482/10/2/prepub
